# Seasonal prevalence of malaria vectors and entomological inoculation rates in the rubber cultivated area of Niete, South Region of Cameroon

**DOI:** 10.1186/1756-3305-5-197

**Published:** 2012-09-10

**Authors:** Jude D Bigoga, Ferdinand M Nanfack, Parfait H Awono-Ambene, Salomon Patchoké, Jean Atangana, Vitalis S Otia, Etienne Fondjo, Roger S Moyou, Rose GF Leke

**Affiliations:** 1 Laboratory for Vector Biology and control, National Reference Unit for Vector Control, The Biotechnology Center, Nkolbisson- University of Yaounde I, P.O. Box 3851-Messa, Yaounde, Cameroon; 2 Faculty of Science, Department of Biochemistry, University of Yaounde I, P.O. Box 812, Yaounde, Cameroon; 3 Organization de Coordination pour la lutte contre les Endemies en Afrique Centrale (OCEAC), Yaounde, Cameroon; 4 Ministry of Public Health, National Malaria Control Program, P.O. Box 14386, Yaounde, Cameroon; 5 Institute of Medical Research and Study of Medicinal Plants (IMPM), Ministry of Research and Innovation (MINRESI), Yaounde, Cameroon; 6 Faculty of Medicine and Biomedical Sciences, University of Yaounde I, Yaounde, Cameroon

**Keywords:** Malaria, Anopheles vectors, Transmission, Rubber cultivation, Cameroon

## Abstract

**Background:**

Development of large scale agro-industries are subject to serious environmental modifications. In malaria endemic areas this would greatly impact on the transmission paradigm. Two cross-sectional entomological surveys to characterize the * Anopheles * fauna and their entomological inoculation rates were conducted during May 2010 (peak rainy season) and December 2010 (peak dry season) in the intense rubber cultivated area of Niete in southern forested Cameroon.

**Methods:**

Mosquitoes were sampled by night collections on human volunteers, identified morphologically and members of the *Anopheles gambiae* complex further identified to species and molecular form. Parity status was determined following the dissection of the ovaries. * Plasmodium falciparum * circumsporozoite antigen indices were estimated after the identification of CS antigen by ELISA and the average entomological inoculation rates determined.

**Results:**

A total of 1187 *Anopheles* was collected, 419 (35.3%) in the rainy season and 768 (64.7%) in the dry season. Species found were the M molecular form of *An. gambiae s.s* (66.8%), * An. ziemanni * (28.3%), * An. paludis * (4.7%), * An. smithii * (0.2%). * An. gambiae * M-form was the principal species in the dry (56.2%) and wet (86.2%) seasons. Average overall entomological inoculation rate for the malaria vectors varied between the dry season (1.09 ib/p/n) and the rainy season (2.30 ib/p/n).

**Conclusions:**

Malaria transmission in Niete occurs both in the dry and rainy season with the intensities peaking in the dry season. This is unlike previous studies in other areas of southern forested Cameroon where transmission generally peaks in the rainy season. Environmental modifications due to agro-industrial activities might have influenced vector distribution and the dynamics of malaria transmission in this area. This necessitates the possible implementation of control strategies that are related to the eco-geography of the area.

## Background

Malaria is the most serious vector borne disease known to mankind and is tightly intertwined with poverty. Most poor countries depend on agro-industry for well-being. Nevertheless, large scale agricultural schemes are subject to serious environmental modifications, which would directly or indirectly impact on the vector species composition, their distribution and consequently the transmission paradigm [1-5]. Although all of Cameroon is endemic for malaria, the level of endemicity greatly varies between the various eco-epidemiological zones depending on the vector species present and the permissiveness of the environment to support their breeding [6-11]. Therefore, the efficacy of anti-vector control for malaria would require requisite baseline information such that measures taken are readily adaptable to the local eco-epidemiological situation [12,13]. At least 45 *Anopheles* species have been reported in Cameroon, 14 of which are implicated in human malaria transmission with varying efficiencies. The major vectors include *Anopheles gambiae* s.s, *An. arabiensisAn. funestus, An. nili* and *A., moucheti*[8,14].

Malaria control in Cameroon relies principally on anti-vector intervention using long lasting insecticidal nets (LLINs) [13,15], and mainly through large-scale campaign and free distribution of the nets. Although such intervention in many areas has been preceded by the acquisition of substantial baseline entomological data ascertaining the anopheline species composition and their degree of involvement in malaria transmission, there has been very little of such evaluation in Niete for several years [16]. Situated within the dense equatorial forest zone of Cameroon where transmission is known to be perennial [13,17], Niete has been occupied principally by the rubber (*Fiscus elastica*) plantation of Hévéas du Cameroun (HEVECAM) since the early 1970s. This agro-development scheme has exerted serious environmental alteration through urbanization, population growth and deforestation, which is thought to have affected the anopheline species composition, and behaviour, and consequently the dynamics of malaria transmission in this locality. Thus, it is important prior to implementation of any anti-vector intervention strategies to acquire substantial baseline entomological data from the diverse eco-geographic zones of Cameroon, especially in areas where very little or nothing is known concerning the malaria vectors and the extent of their involvement in malaria transmission.

Worldwide, efforts to eliminate and eradicate malaria warrant an integrated approach involving antivector-interventions, chemotherapy and vaccination [18]. However, with the continuous evolution and spread of drug resistant forms of the parasite and resistance to insecticides in the major vector species, there is need to evaluate other forms of new drugs or drug combinations to identify the best treatment. Also vaccine molecules are becoming available and need to be tested in the field. Such trials require a well a characterized field site and stable population. Consequently, as part of an ongoing study to acquire baseline data for the preparation of Niete for future malaria drug/vaccine trials in Cameroon, this study describes the anopheline fauna and their contribution to malaria transmission during the dry and rainy seasons in the rubber cultivated area of Niete in the Ocean Division, South region of Cameroon.

## Methods

### Study area

**Figure 1:**
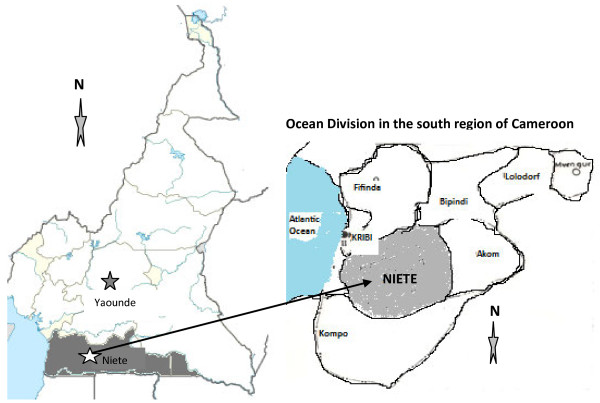
Map of Cameroon showing the Location of the study site (Niete).

This study was carried out in May 2010 (peak rainy season) and in December 2010 (peak dry season) in the rubber cultivated area of Niete (N02°43.508; E10°04.118; alt: 9 m) in the Southern region of Cameroon. Niete is practically occupied by the rubber plantation of HEVECAM (Hévéas du Cameroun), which was created in 1975 and employs over 6000 workers. The plantation spreads over 40000 hectares, about 40% of the forest. Mainly plantation workers live there, with farming being the major economic activity. The climate is essentially equatorial with four seasons; two rainy seasons (late March to June and September to early November) alternating with two dry seasons (late November to early March and July to August). It has an average annual rainfall of 1500–2000 mm (averaging 50 mm in the month of December and 2500 mm in May), an average temperature of 25 °C and relative humidity of 88% [16]. The vegetation is composed of dense forest studded with marshy areas that are prolific for mosquito breeding. There are two main streams, namely, Nye’été and Nlongo. Figure 1 depicts the map of the study site.

### Ethical considerations

This study was conducted within the framework of a larger ongoing study to acquire baseline malaria data in order to help establish a cohort and site for future drug/vaccine trials in Cameroon. An ethical clearance document was obtained from the National ethics committee of Cameroon. Consent of household heads was sought prior to using the house for mosquito collection. Participation in mosquito collection was strictly voluntary and only those adequately trained on the collection process were retained. All collectors were given anti malaria prophylaxis based on Artesunate/Amodiaquine.

### Field sampling of adult mosquitoes

Human landing collections were performed during five consecutive nights from 06:00 pm-06:00 am each season. Mosquitoes were collected indoors and outdoors in three randomly selected houses (at least 50 m apart) and rotating between houses at different locations in each village each night. A team of two trained volunteers per house; one sitting inside the house and the other on the veranda collected female mosquitoes as they landed on exposed lower limbs, making a total of 30 human-nights per season. Thus, a total of 60 human nights for the two seasons. The mosquitoes were sorted by genus and the anophelines were identified morphologically using the keys of Gillies and De Meillon [19] and Gillies and Coetzee [20]. The ovaries of a proportion of unfed mosquitoes were dissected and the tracheoles examined for parity determination [21]. All dissected and undissected mosquitoes were individually stored desiccated in tubes containing silica gel for subsequent laboratory analyses.

### Laboratory processing of anophelines

A consecutive random sample of 120 members of the *An. gambiae* complex (60 per season) was further identified to species and molecular forms level. Briefly, genomic DNA from the legs and wings of randomly selected mosquitoes of the *Anopheles gambiae* complex was extracted as described by Collins *et al*. [22]. The DNA was re-suspended in 25 μl sterile TE-buffer (10 mM Tris–HCl pH 8.1, 1 mM EDTA) and used to identify the siblings of *An. gambiae* by the standard ribosomal DNA polymerase chain reaction technique [23]. Presence of molecular M and S forms of *An. gambiae* s.s. was ascertained by restriction fragment length polymorphism (RFLP) PCR analysis of the X-linked ribosomal DNA [24]. The head-thorax portion of each mosquito was tested for *P. falciparum* circumsporozoite antigen by ELISA [25,26]. The infection rates were calculated and the entomological inoculation rate determined.

### Data analysis

Entomological parameters considered were : 1) man biting rate (ma), calculated as the average number of bites received per person per night of collection; 2) infection rate, measured as the proportion of mosquitoes positive for *P. falciparum* circumsporozoite antigen by ELISA; 3) Parity rate, measured as the ratio of parous mosquitoes to the total of parous and nulliparous mosquitoes dissected; 4) Entomological inoculation rate (EIR), calculated as the product of the man biting rate and circumsporozoite antigen rate. Data were analyzed in R statistics software version 2.8 and the Kruskal Wallis test was used to compare means at 95% confidence interval.

## Results

### Mosquito composition and Anopheles biting habits

A total of 3247 mosquitoes were collected during 60 human nights in both the wet and dry seasons. Genera found were *Culex* (62.2%), *Anopheles* (36.6%), *Aedes* (1%) and *Coquillettidia* (0.2%). While Culex (79.6%) dominated the mosquito fauna during the rainy season, *Anopheles* was the most abundant in the dry season (61.95%). A total of 1187 *Anopheles* were collected: 419 (35.5%) in the rainy season and 768 (64.7) in the dry season. *An. gambiae* s.l. was the predominant species in both the dry (56.2%) and the wet (86.2%) seasons. While *An. paludis* (4.7%) and *An. smithii* (0.08%) were found only during the rainy season, *An. ziemanni* (28.3%) was found only during the dry season (Table 1). Molecular analysis of the *An. gambiae* complex siblings revealed only the presence of *Anopheles gambiae* s.s, M molecular form.

**Table 1:** Distribution of mosquito fauna in the rubber cultivated area of Niete by season

**Mosquito species**	**Wet season**	**Dry season**	**Total**	**Proportion total* Anopheles *(%)**	**Proportion total mosquitoes (%)**
*An. gambiae s.l.*	362	432	794	66,89%	24,45%
*An. paludis*	56	0	56	4,72%	1,72%
*An. smithii*	1		1	0,08%	0,03%
*An. ziemanni*	0	336	336	28,31%	10,35%
Total Anopheles	**419 (35.5%)**	**768 (64.65%)**	**1187**	**100,00%**	**36,56%**
*Culex sp*	1640	382	2022	/	62,27%
*Aedes sp*	6	28	34	/	1,05%
*Coquilettidia sp*	1	3	4	/	0,12%
Total other culicidae	**1647**	**413**	**2060**	**/**	**63,44%**
**Total mosquitoes**	**1857**	**697**	**3247**	**/**	**100%**

**Figure 2:**
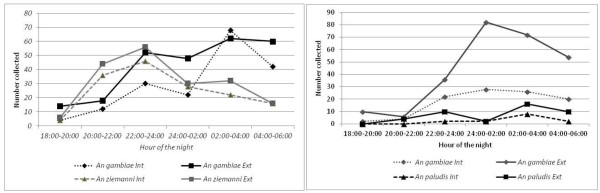
Seasonal variation in the indoor and outdoor biting cycle of anophelines in the rubber cultivated area of Niete.

*Anopheles gambiae* was the most aggressive species with a bite rate of 12.07 bites/person/night (b/p/n) in the rainy season and 14.4 b/p/n in the dry season. *An. ziemanni* had higher bite rates (11.2 b/p/n) compared to *An. gambiae*. The difference, however, was not significant (p > 0.05) (Table 2). The night human biting cycle (Figure 2) showed peak biting hours for *An. gambiae* in the dry season between 02:00 and 04:00 respectively for indoor and outdoor biting females and in the rainy season between 24:00 and 02:00. With the exception of *An. gambiae* that was mostly exophagous during the rainy season (P < 0.05), the other *Anopheles* species showed no preference for the location of biting (P > 0.05).

**Table 2:** Parity rates of the malaria vectors by season

***Anopheles*species**	**Rainy season**		**Dry season**		**Total (dry and rainy season)**	
	**n**	**Parous rate [95% CI]**	**n**	**Parous rate [95% CI]**	**n**	**Parous rate [95% CI]**
*An. gambiae.*	161	91.30	166	76.86	327	81.35
		[86.95 -95.65]		[70.44-83.28]		[77.12-85.88]
*An. paludis*	26	88.46	-	-	26	88.46
		[77.86-99.04]				[77.86-99.04]
*An. ziemanni*	-	-	103	64.08	103	64.08
				[54.81-73.35]		[54.81-73.35]
**Total**	**187**	**90.91**	**269**	**68.77**	**456**	**77.85**
		**[86.79-95.03]**		**[63.23-74.31]**		**[74.04-81.66]**

**n**: Number collected; **CI**: confidence interval.

### Parity rates

Table 2 shows the estimated parous rates of the *Anopheles* species during the two seasons. Although numerically more *Anopheles gambiae* laid eggs at least once in the rainy season (91.3%) compared to the dry season (76.86%), this difference was not significant (χ2 = 1,90; df = 2; P > 0.05). There were also more parous than nulliparous *An. paludis* found only during the rainy season (88.5%) and for *An. ziemanni* (64.08%) collected only during the dry season

### Infection rates and entomological inoculation rates

A total of 1179 *Anopheles* mosquitoes were examined for *P. falciparum* circumsporozoite antigen by ELISA during both seasons of which 103 (8.76%) were infected. The overall average infection rate was 15% (n = 419) during the rainy season and 17% (n = 768) in the dry season. Of these, there were 90.2% infections in *An. gambiae*, 3.92% in *An. paludis* and 5.88% in *An. ziemanni*. Table 3 depicts the man biting rates, circumsporozoite antigen (CSA) rates and the entomological inoculation rates (EIR) by vector species. Apart from the lone *An. smithii,* which was not infectious, all the other anophelines carried *P. falciparum* circumsporozoite antigen. *An. gambiae* recorded the highest infection rates during both seasons with 8% (n = 354) in the rainy season and 14% (n = 432) in the dry season. Of the 56 *An. paludis* collected and tested during the dry season, 4 (7%) were positive, while 3% (10/336) of *An. ziemanni* found only during the dry season were positive.

**Table 3:** Daily mean biting rate (ma) Circumsporozoite antigen (CSA) indices and entomological inoculation rates (EIR) of vectors by season

***Anopheles*species**	**Rainy season**					**Dry season**				
	**No collected**	**ma**	**Tested for CSA**	**CSA index**	**EIR [95% CI]**	**No collected**	**ma**	**Tested for CSA**	**CSA index**	**EIR [95% CI]**
***An. gambiae s.l.***	362	12.07	354	0.08 (±0.03)	0.95 [0.61-1.29]	432	14.4	432	0.14 (±0.03)	2.05 [1.62 -2.48]
***An. paludis***	56	1.87	56	0.07 (±0.06)	0.13 [0.02-0.24]	0	0	0	0	0
***An. ziemanni***	0	0,00	0	0	0.00	336	11.2	336	0.03 (±0.02)	0,32 [0.1-0.52]
***Total***	**418**	**13.93**	**411**	**0.08 (±0.03)**	**1.11 [0,70 -1.53**	**768**	**25,6**	**768**	**0.09 (±0.02)**	**2.30 [1.79-2.82]**

Overall, the estimated average entomological inoculation rates were two-fold higher during the rainy season with 0.58 infective bites per person per night (ib/p/n) compared to the dry season (0.29ib/p/n). The highest inoculation rate was recorded for *Anopheles gambiae* averaging 0.79ib/p/n for the two seasons. This, however, varied between the seasons for this species with 0.95 ib/p/n during the rainy season and 2.05 ib/p/n during the dry season. The number of infective bites for *An. paludis* was 0.13ib/p/n during the rainy season while that for *An. ziemanni* was 0.32 ib/p/n in the dry season.

## Discussion

The results show that *An. gambiae s.s., An. paludis* and *An. ziemanni* are the malaria vectors in the studied area as they were found to contain *P. falciparum* circumsporozoite antigen. This study further confirms the role of *An. gambiae* as the major malaria vector in the southern forested areas of Cameroon and Western Africa and more so in areas of intense large scale agricultural activities as reported previously [1,12,15,27-30]. The observed night biting cycles of the anophelines peaked generally between 10:00 pm and 02:00 am and did not deviate from the prototype described earlier by Gillies and De Meillon [19,29]. The huge abundance of non malaria vector mosquitoes both in the dry and wet season pinpoints the discriminating level of nuisance they might cause in the locality.

Unlike previous observations in the southern forested areas of Cameroon, more *Anopheles* mosquitoes especially *Anopheles gambiae,* were collected during the dry season compared to the rainy season [13,31]. This is presumably due to the fact that sampling was performed during the peak rainy month of the year, when most of the breeding sources were constantly flushed, thus carrying away most of the aquatic stages. However, the much reduced water beds of the Nye’ete and Nlongo rivers happen to be prolific sites for vector breeding during the dry season.

Although the roles of *An. paludis* and *An. ziemanni* are confirmed here as secondary vectors of malaria transmission in Cameroon [8,32], these species play significantly important roles in malaria transmission and would therefore significantly maintain and extend the duration of transmission in this locality. The lone *An. smithii* collected was not infectious. Though present in Cameroon, this species has never been associated with human malaria transmission [32].

Akin to earlier observations in the rubber plantations of coastal south western Cameroon, the high parous rates of the vectors in Niete indicates that there is accumulation of an ageing adult *Anopheles* population in this locality over time [13]. This is epidemiologically dangerous, as the vectors would exhibit higher feeding frequencies on humans and being able to transmit the malaria parasite over and again [33]. This increase in the number of ageing mosquitoes is suggestive that the anti-vector intervention measures implored might not be efficacious and that the vectors might have simply developed resistance to insecticides [34], making it possible for them to survive longer with increased chances of transmitting malaria parasites more than once.

The CSA rates were higher in *An. gambiae* than the other vectors. This is not improbable and is in support of previous studies in the southern forested region of Cameroon [6,13,17,35]. The fact that transmission occurs both during the dry and wet seasons might be indicative of a perennial pattern. However, the overall entomological inoculation rates varied seasonally with higher rates in the dry season. This is contrary to other earlier studies in the southern forested Cameroon where peak transmission generally parallels peak rainfall. As shown, persons living in this locality if not protected, stand the risk of getting a daily infective mosquito bite of 1.11 during the rainy season and 2.30 in the dry season.

## Conclusion

This study depicts that malaria transmission in Niete occurs during both the rainy and dry seasons with the intensity varying seasonally with the vector species involved. Consequently, it provides preliminary baseline information needed for planning and implementation of anti-vector control measures amenable to the local eco-epidemiological situation.

## Competing interests

The authors declare that they have no competing interests.

## Authors’ contributions

JDB, RSM and RGFL planned the study design. FN, SP, PHA and JDB performed field activity, laboratory work and analyzed the data, JDB and FN drafted the manuscript. PHA, RGFL, RM and EF provided substantial improvement of the manuscript. All authors approved the final version of the manuscript.
